# A user evaluation of speech/phrase recognition software in critically ill patients: a DECIDE-AI feasibility study

**DOI:** 10.1186/s13054-023-04420-x

**Published:** 2023-07-10

**Authors:** M. Musalia, S. Laha, J. Cazalilla-Chica, J. Allan, L. Roach, J. Twamley, S. Nanda, M. Verlander, A. Williams, I. Kempe, I. I. Patel, F. Campbell-West, B. Blackwood, D. F. McAuley

**Affiliations:** 1 Critical Care Unit, Lancashire Teaching Hospitals NHS Foundation Trust, Preston, UK; 2 Faculty of Biology Medicine and Health, University of Manchester, Manchester, UK; 3 Liopa, Northern Ireland Science Park, Belfast, UK; 4 Wellcome-Wolfson Institute for Experimental Medicine, Queens University Belfast, Belfast, UK; 5 Faculty of Health and Care, University of Central Lancashire, Preston, UK

**Keywords:** Tracheostomy, SRAVI, Lip reading, Speech, Accuracy

## Abstract

**Objectives:**

Evaluating effectiveness of speech/phrase recognition software in critically ill patients with speech impairments.

**Design:**

Prospective study.

**Setting:**

Tertiary hospital critical care unit in the northwest of England.

**Participants:**

14 patients with tracheostomies, 3 female and 11 male.

**Main outcome measures:**

Evaluation of dynamic time warping (DTW) and deep neural networks (DNN) methods in a speech/phrase recognition application. Using speech/phrase recognition app for voice impaired (SRAVI), patients attempted mouthing various supported phrases with recordings evaluated by both DNN and DTW processing methods. Then, a trio of potential recognition phrases was displayed on the screen, ranked from first to third in order of likelihood.

**Results:**

A total of 616 patient recordings were taken with 516 phrase identifiable recordings**.** The overall results revealed a total recognition accuracy across all three ranks of 86% using the DNN method. The rank 1 recognition accuracy of the DNN method was 75%. The DTW method had a total recognition accuracy of 74%, with a rank 1 accuracy of 48%.

**Conclusion:**

This feasibility evaluation of a novel speech/phrase recognition app using SRAVI demonstrated a good correlation between spoken phrases and app recognition. This suggests that speech/phrase recognition technology could be a therapeutic option to bridge the gap in communication in critically ill patients.

**What is already known about this topic:**

Communication can be attempted using visual charts, eye gaze boards, alphabet boards, speech/phrase reading, gestures and speaking valves in critically ill patients with speech impairments.

**What this study adds:**

Deep neural networks and dynamic time warping methods can be used to analyse lip movements and identify intended phrases.

**How this study might affect research, practice and policy:**

Our study shows that speech/phrase recognition software has a role to play in bridging the communication gap in speech impairment.

**Supplementary Information:**

The online version contains supplementary material available at 10.1186/s13054-023-04420-x.

## Introduction

Communication for individuals with speech impairments is a challenging issue in the hospital setting [1]. This is predominantly so for those that have acutely lost their ability to verbalise, a problem commonly encountered in the critical care setting. This includes patients with neurological pathologies, vocal cord injury, laryngectomies, head and neck tumours and more commonly tracheostomies. This can lead to loss of autonomy and frustration for patients as well as those caring for them [1]. Approximately 14,000 critical care patients will undergo a tracheostomy annually in the UK, and 58,800 in the US [2, 3].

Studies have shown increased incidences of agitation, need for sedation and the presence of adverse events where communication needs are not met, further highlighting the importance of effective communication tools within this cohort of vocally impaired patients [4]. Commonly employed methods have included simple tasks such as ‘mouthing’ words, gestures, nodding ‘yes’ or ‘no’, writing on paper, visual charts, alphabet boards and use of eye gaze boards amongst others [5]. Various limitations to all of these methods exist, from a simple inability to lip read or understand gestures and patients being too tired or weak to write. Furthermore, the simple closed questions and discrete nature of visual charts do not allow any room for the exact expression of a patient’s needs [6–8]. Alphabet boards are also simply too time consuming to be deemed practical in a dynamic critical care setting [9]. The incorporation of speaking valves into tracheostomy tubes has proved invaluable, but also requires patients to be at an advanced point of their respiratory weaning and require expertise [8]. Currently, there is no communication tool in which all patients' needs are met. Speech/phrase recognition technology could play a role in fulfilling patients’ communication needs both in the Intensive Care Unit (ICU) and wider hospital setting. Using the DECIDE-AI guidelines for healthcare technologies, we demonstrate the feasibility of speech/phrase recognition technology for speech impairment in critically ill patients [10].

## Methods

### Design

This was a prospective development study. The evaluation was a collaborative process by Lancashire Teaching Hospitals NHS Foundation Trust, Liopa and Queen’s University Belfast. It was funded by Innovate UK.

**Fig. 1:**
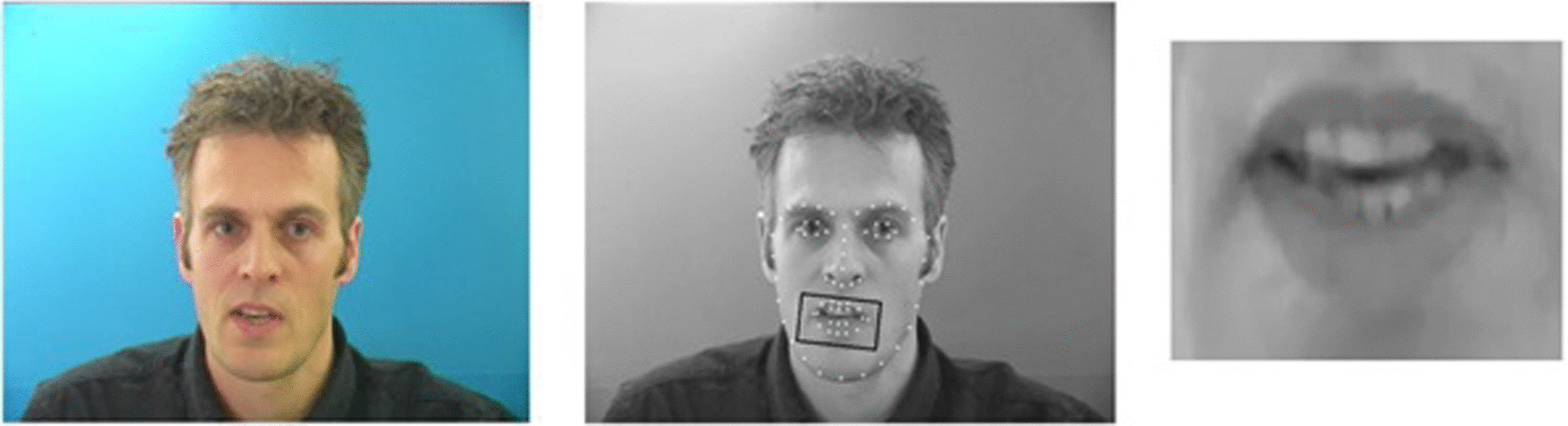
Illustration of automatic detection and extraction of the lip region from an image

A handheld android/iOS device with internet access (Wi-Fi or 4G telephone network) was used. The app was downloaded onto the devices and initial calibrations were completed for each user, ensuring sufficient visibility via the identification and extraction of each individual’s lip region (Fig. 1). The selected phrase lists were able to be viewed on all devices, although were mostly viewed on a larger laminated sheet, allowing easier reference for the patient.

**Fig. 2:**
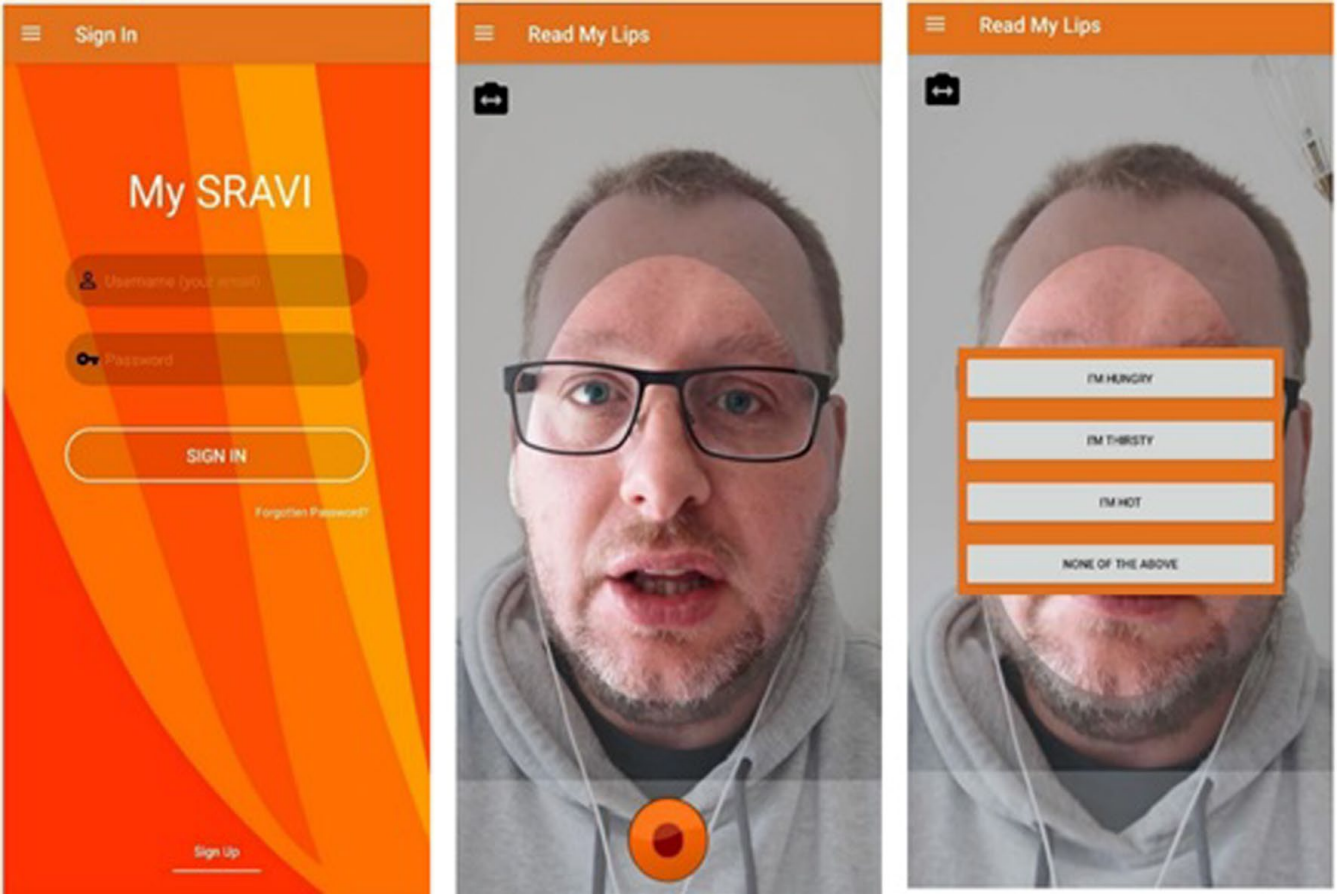
Speech/phrase recognition app for voice impaired (SRAVI); *Login (left), recording (middle) and result (right) screens in the SRAVI app*

Patients were given a basic tutorial on how to use the app. Referring to the list of supported phrases (Additional file 1: Fig. S1), patients attempted to speak a phrase of their choice, allowing SRAVI to return a list of three possible results ranked in order of likelihood, from first to third (Fig. 2). Optimal distance from the camera was determined by having the face enclosed within the highlighted oval face shape within the app (Fig. 2). The patient would then confirm the correct phrase if present amongst the available choices, or indicate if the choice was not present. The available phrases were reviewed and refined during the recruitment phase in accordance with user feedback. Patients were allowed to use the app as able.

### Technical description of SRAVI

**Fig. 3:**
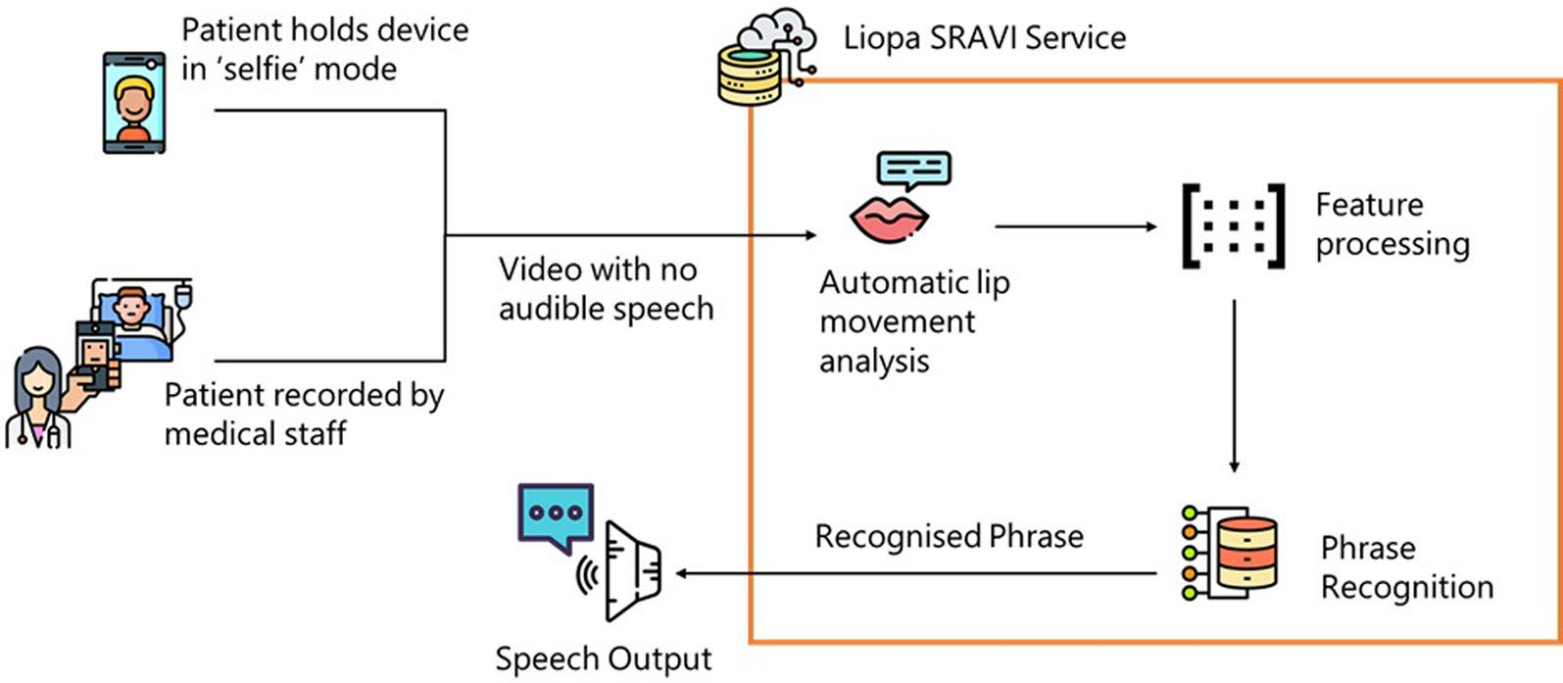
Speech/phrase recognition app for voice impaired (SRAVI) Architecture

Lip movements are analysed by tracking points on the face and aligning the images so that the face is relatively still, and the lips are clearly visible. Features, such as appearance, velocity and acceleration, are extracted from the video of the lip movements and algorithmically evaluated to determine what phrase was uttered (Fig. 3). SRAVI analyses each frame of a video by first performing a 64-point facial identification. Using facial landmarks, the image is cropped to a rectangular box in the mouth region, converted to grayscale and fed into the analysis (Fig. 1).

Two methods for recognising speech/phrase, referred to as the dynamic time warping (DTW) method and the deep neural network (DNN) method, respectively, are used. The DTW method is computationally more efficient, has been adapted to run on a mobile device and can learn from the user. DTW compares a set of previous time series created from videos of a subject correctly mouthing all of the phrases that the system is trained to recognise. After calculating the distances, we determine the warping path. The sum of the minimum distances gives us a measure of similarity.

For maximum benefit, however, DTW requires the user to provide training data to calibrate the system, and for patients in ICU, this is not always practical. To circumvent this, a method for using DTW without the user having to provide training data has been developed. The DNN method uses state-of-the-art artificial intelligence techniques to create a digital representation of lip movements with over 5,00,000 samples from thousands of different speakers. This digital representation is referred to as a model and it is created, or trained, by learning from these examples. The DNN model is more general, since it has amalgamated all the different speakers in the training samples. However, as covered in the discussion section, it may not recognise lip movements that differ significantly from these samples. Additionally, patients in this study were not used to train the model and therefore would not iteratively affect the accuracy in this study.

## Setting and participants

The study was undertaken at the Lancashire Teaching Hospitals NHS Foundation Trust, UK. Eligibility criteria included recent admission to the critical care unit, underlying pathology or interventions that impaired speech, understanding of English, the absence of cognitive impairment that would hinder ability to use the application, and ability to follow commands. Patients who were sedated, too unwell or showing marked cognitive impairment were excluded. Patients who fulfilled eligibility criteria were approached.

The evaluation was a collaborative process by Lancashire Teaching Hospitals NHS Foundation Trust, Liopa and Queen’s University Belfast. It was funded by the UKRI Innovate UK Digital health technology catalyst round 3 feasibility studies process.

## Results

20 participants were initially recruited, however 6 were withdrawn due to clinical deterioration, early termination of critical care needs or a personal desire not to participate further. We were able to use SRAVI on 14 participants (3 female and 11 male). The ages were distributed as follows: 3 were between 18 and 39 years old, 6 between 40 and 59 years, and 5 were above 60 years. 12 participants were Caucasian, and 2 were Asian. Over 7-month period, 616 valid patient video recordings were taken.

All recordings were evaluated by both DTW and DNN processing methods, with each method returning a list of three potential phrases. The first phrase returned was the most likely result based on lip movement and was reported as ‘rank one ‘, with the second and third as rank two and rank three. Each rank phrase determined was irrespective of the phrase of other ranks. A breakdown of results per patients is shown below indicating number of phrase identifiable recordings and the recognition accuracy using both DTW and DNN methods, i.e. total recognition accuracy (Table 1).

**Table 1:** Number of recordings, number of phrase identifiable recordings and total recognition accuracy of SRAVI for each of the 14 patients

Patient ID	Total recordings	Phrase identifiable recordings	SRAVI accuracy
P1	6	6	100%
P2	17	14	80%
P3	3	3	100%
P4	4	2	50%
P5	155	144	93%
P6	31	17	55%
P7	17	11	65%
P8	61	40	66%
P9	203	189	93%
P10	19	0	N/A
P11	30	24	80%
P12	20	20	100%
P13	46	46	100%
P14	4	0	N/A

For each patient, the percentage of patient-identifiable recordings (Table 1) and the recognition accuracy for those samples is shown. A patient identifiable recording is a clear recording of a patient that can be automatically analysed. Of particular note are patients 10 and 14, where none of the recording phrases were identifiable. Patient 14 only recorded 4 videos and was feeling too unwell to continue. Patient 10 recorded 19 videos and was lying in a slightly elevated prone position, which made positioning of the camera difficult. All the recordings were at a severe angle, and the lip region was not clearly visible.

**Fig. 4:**
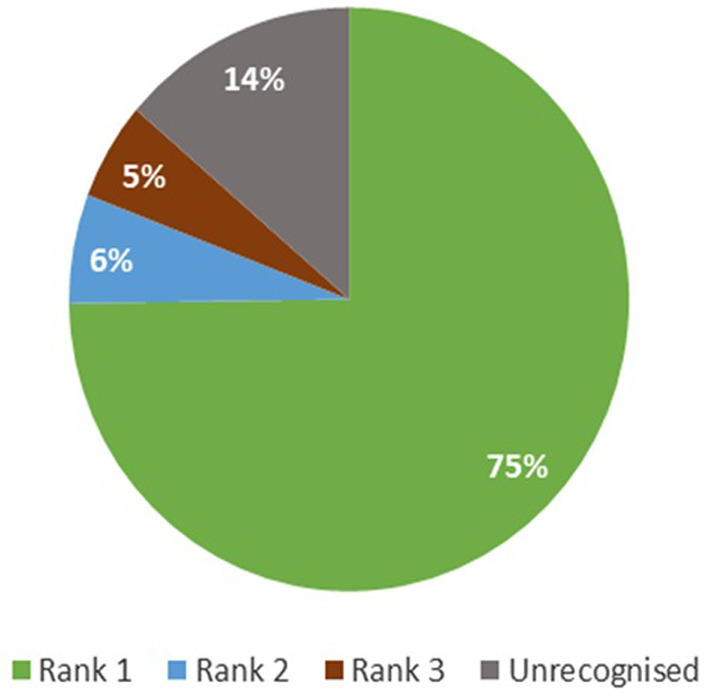
Recognition accuracy for the deep neural network (DNN) method

**Fig. 5:**
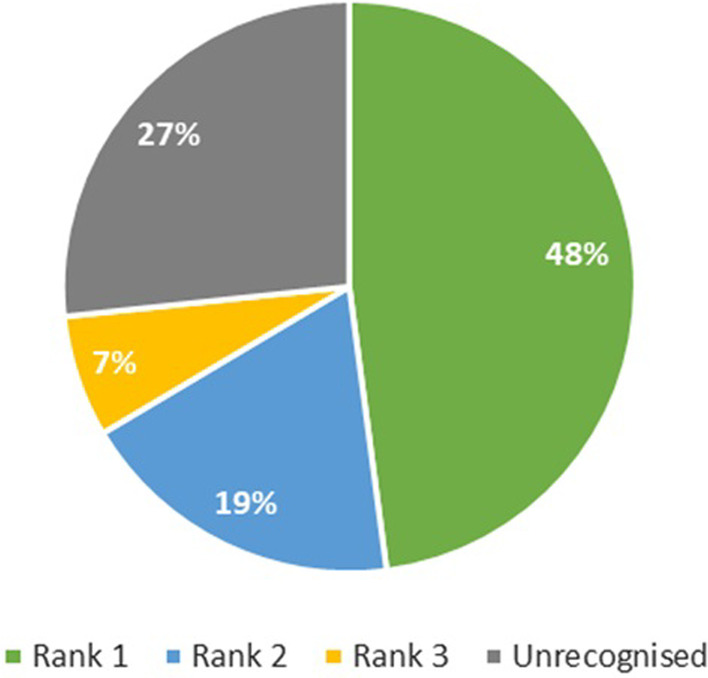
Recognition accuracy for the dynamic time warping (DTW) method

The rank 1 recognition accuracy of the DNN method is 75%, whilst the total recognition accuracy was 86%, accounting for all three ranked responses (Fig. 4). The DTW method performed markedly worse, with a rank 1 accuracy of 48% and a total recognition accuracy of 74% (Fig. 5).

The DTW method performed markedly worse, with a rank 1 accuracy of 48% and a total recognition accuracy of 74% (Fig. 5).

## Discussion

Over a 7-month period, a total of 657 videos from 14 different participants were made. The overall results revealed a total recognition accuracy of 86% for DNN and 75% for DTW (Additional file 1: Fig. S2). This high accuracy rating indicates the suitability of speech/phrase recognition technology as a therapeutic option to bridge the gap in communication in critically ill patients.

The factors which can contribute to errors in accuracy are discussed below:

### Environmental factors

Variations in brightness and visibility in different environments proved challenging for automated speech/phrase recognition and, in some cases, failed to identify the phrases mouthed. Examples of illumination variations are shadows across the face, differences between natural and artificial light and reflections in eyeglasses. In the ICU setting, environmental factors such as medical equipment, e.g. tubes, ventilators, neck supports, nasogastric tubes and facial dressings all proved to affect the use of SRAVI. One or more of these environmental factors were present in over half of our patient sample, which proved to be especially challenging during the study.

### Speaker factors

**Fig. 6:**
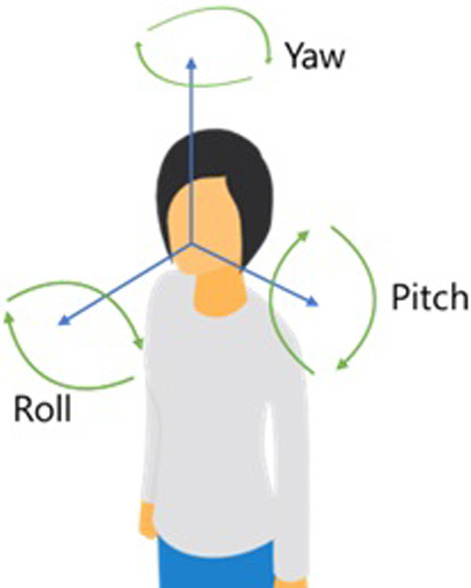
Illustration of roll, pitch and yaw and the six degrees of freedom

Considering the relation between the camera and patient, variations in pose were attributable to the results observed. The unconstrained patient would face the camera at varying angles. This introduces the concepts of roll, pitch and yaw; scale and the six degrees of freedom a person/object has in space (Fig. 6). The pose angle and distance from the camera both affect the relative size of the face and lips in the image. Subsequently, such factors can determine the speech/phrase recognition capability and accuracy of results. Most users reported better results when the patient was sat upright, as opposed to lying down or at an angle. Although, this patient position is somewhat rarely achievable in the critical care setting.

### Technical parameter factors

A number of parameters involved in SRAVI all impacted on overall performance, including the following: pixel resolution of recorded video, video frame, bitrate of the encoded video, size of the video, quality profile of the recording and quality of the camera sensor. All of these are adjustable but must balance performance with the user experience. Higher quality videos will enhance performance, but are larger and take longer to transmit and process. Staff also reported challenges with poor connectivity making it difficult to carry out trial sessions. This may have been an isolated, geographical connectivity issue within the critical care unit at LTHTR; however, connectivity must be accounted for before using the SRAVI app in any location.

## Staff

Staff were approached and briefed on what SRAVI was and how we hoped to assess its use in the critical care patients with speech impairments. Those that volunteered to take part had training done by the clinical and technical team to ensure their competence. They also had a chance to use the App themselves and gave input as to patient and staff factors, they felt were of use. Due to the critical nature of the patients, staff were predominantly required to aid the patients for use of the app.

## Limitations

The study recruited a small number of participants in its initial phase. There were also variations in the number of recordings made by individual patients because of personal, environmental and technological factors.

## Conclusion

This pilot study of a novel speech/phrase recognition app (SRAVI) demonstrated a good correlation between spoken phrases and app recognition. Various patient and environmental factors have been identified as potential challenges to the use of SRAVI which need to be addressed. The SRAVI pilot study will hopefully pave the way for further research to develop the effectiveness of the speech/phrase recognition technology using SRAVI. The next steps will include efforts to improve the robustness against illuminations and pose changes, reducing latency for the end user, formal training of staff, recruitment of more patients to the SRAVI trial and assessing the possibility of moving the processing to the mobile handset.

## Supplementary Information

**Additional file 1.** **Supplementary Figure 1.** Speech recognition app for voice impaired (SRAVI) phrase list. **Supplementary Figure 2.** Comparison of deep neural networks (DNN) vs dynamic time warping (DTW) for phrase recognition.

## Data Availability

Data sharing for additional information will be considered by the group based on written requests to the corresponding author.
