# Supplementary material for: A user evaluation of speech/phrase recognition software in critically ill patients: a DECIDE-AI feasibility study
Source: Crit Care. 2023 Jul 10;27:277. doi: 10.1186/s13054-023-04420-x (PMC10332046; doi:10.1186/s13054-023-04420-x)
Supplement: Supplementary file 1 — Additional file 1. Supplementary Figure 1. Speech recognition app for voice impaired (SRAVI) phrase list. Supplementary Figure 2. Comparison of deep neural networks (DNN) vs dynamic time warping (DTW) for phrase recognition. [file 13054_2023_4420_MOESM1_ESM.docx]

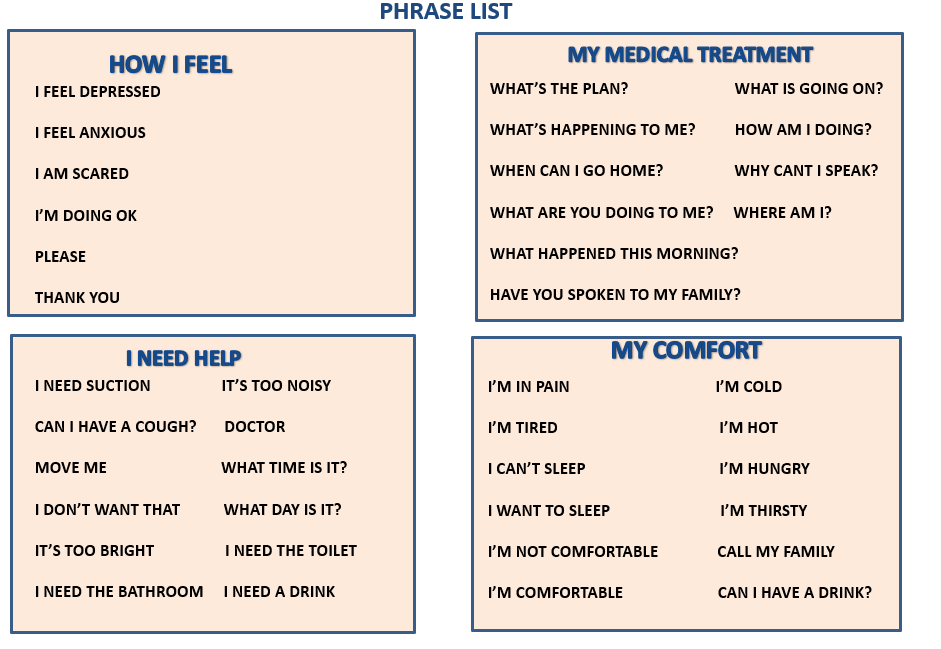


Supplementary Figure 1 – Speech recognition app for voice impaired (SRAVI) phrase list


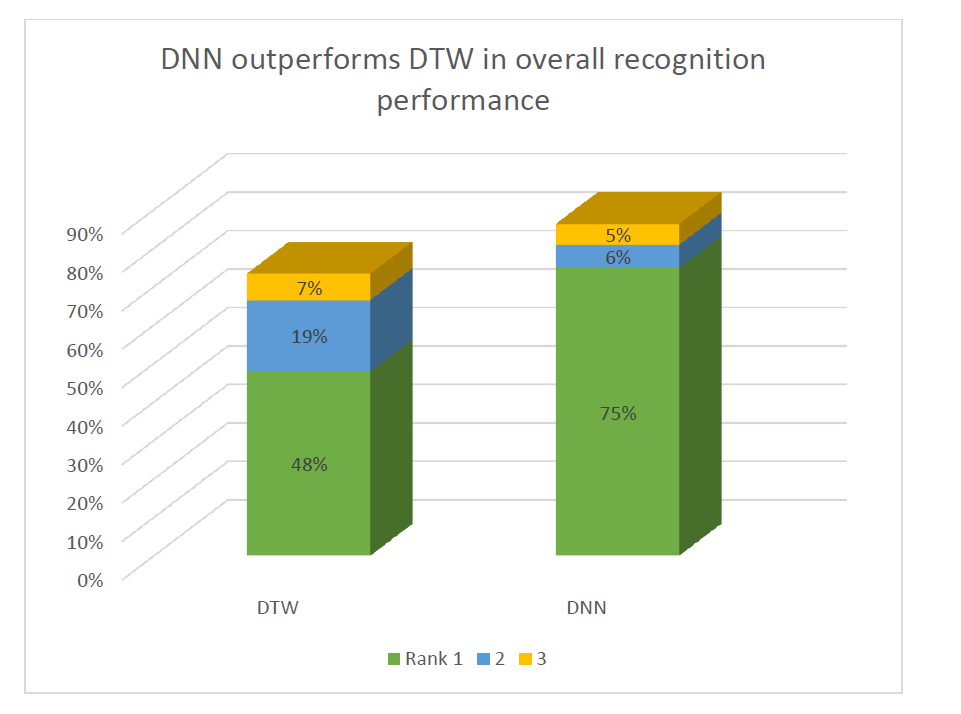


Supplementary Figure 2 – Comparison of deep neural networks (DNN) vs dynamic time warping (DTW) for phrase recognition. Allocation for phrases was set as Rank 1 (green), Rank 2 (blue) and Rank 3 (yellow).
